# Sex-specific transgenerational effects of diet on offspring life history and physiology

**DOI:** 10.1098/rspb.2024.0062

**Published:** 2024-04-17

**Authors:** Tara-Lyn Camilleri, Matthew D. W. Piper, Rebecca L. Robker, Damian K. Dowling

**Affiliations:** ^1^ School of Biological Sciences, Monash University, Melbourne, Victoria 3800, Australia; ^2^ School of Biomedical Sciences, Monash University, Melbourne, Victoria 3800, Australia; ^3^ School of Paediatrics and Reproductive Health, Robinson Research Institute, The University of Adelaide, Adelaide 5005, Australia; ^4^ Department of Biology, University of Oxford, Oxford, Oxfordshire, UK

**Keywords:** transgenerational effects, sucrose, life history, dietary restriction, *Drosophila*

## Abstract

Dietary variation in males and females can shape the expression of offspring life histories and physiology. However, the relative contributions of maternal and paternal dietary variation to phenotypic expression of latter generations is currently unknown. We provided male and female *Drosophila melanogaster* grandparents with diets differing in sucrose concentration prior to reproduction, and similarly subjected their grandoffspring to the same treatments. We then investigated the phenotypic consequences of this dietary variation among the grandsons and granddaughters. We observed transgenerational effects of dietary sucrose, mediated through the grandmaternal lineage, which mimic the direct effects of sucrose on lifespan, with opposing patterns across sexes; low sucrose increased female, but decreased male, lifespan. Dietary mismatching of grandoffspring–grandparent diets increased lifespan and reproductive success, and moderated triglyceride levels of grandoffspring, providing insights into the physiological underpinnings of the complex transgenerational effects on life histories.

## 1. Background

Parental environments may shape the phenotypes of their offspring through non-genetic mechanisms that can be condition-dependent and epigenetic in origin [1–4]. Consequently, when individuals are subjected to environmental heterogeneity prior to reproduction, their exposure to these environments can shape components of fitness in offspring (intergenerational effects F1) and subsequent generations (transgenerational effects F2+) [5–9]. Recent experiments have shown that variation in environmental factors such as predation risk and levels of sexual conflict among parents may give rise to transgenerational effects that differ in magnitude or direction across sexes, and these effects may also be lineage-specific, that is, effects may differ depending on whether they are passed from the dam or sire line [10].

Nutrition is a pervasive and critical source of environmental variation that can shape phenotypes. Variation in macronutrient balance or caloric content can have direct effects on lifespan, fecundity and underlying physiology [11–14]. Studies from diverse species have demonstrated that females and males require different diets to maximize their fitness [15–19]. In both invertebrates and vertebrates, female fitness is maximized on a higher relative protein concentration because high protein facilitates egg production, while higher relative carbohydrate content for males provides fuel for attracting and locating mates [20–24]. Recent studies have also shown dietary-induced intergenerational effects across a variety of species; for example, changes to the sugar content of the parental diets in fruit flies (*Drosophila melanogaster*) [25,26] or dietary fat content in mice (C57BL/6NTac *Mus musculus*) [27] induce phenotypic changes in parents that are transmitted to their offspring. For example, when the sucrose content of both male and female fruit flies was altered, the parental contributions to offspring phenotypes manifested as complex dam-by-sire interactions that were non-cumulative, and furthermore dependent upon the sucrose content of the offspring diet. The results from this study also showed that matching diets between parent and offspring did not confer a fitness advantage to the offspring [11]. It is less clear, however, whether these dietary-mediated parental effects are inherited across multiple generations [10,25,28–31] and if so, whether they are transferred primarily through maternal or paternal lineages or hinge on interactions between both, and whether they generally act to enhance or depress offspring performance in the same way across offspring sexes.

**Figure 1.**
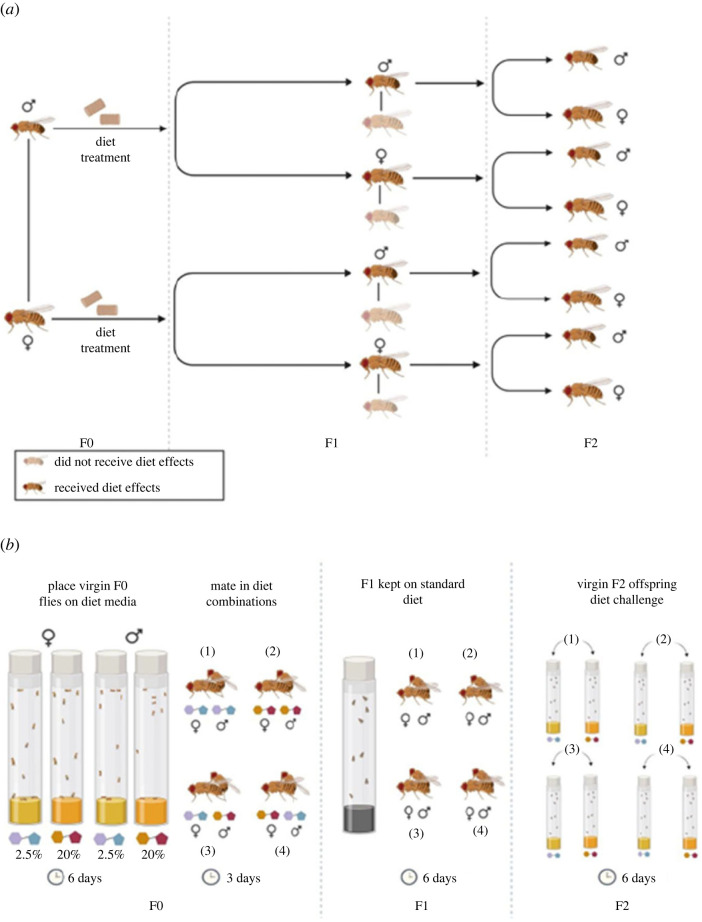
(*a*) Diet effects lineage. Diet treatments were administered to parents of both sexes in the F0; and they were then mated to create the F1 generation. The F1 generation of both sexes received a standard diet and F1 flies of each sex were then mated to *tester* flies (sourced from a stock population outside of the experiment that also received a standard diet). This allowed us to track which F1 sex was passing on the diet effects of the F0 flies to the F2 generation. ‘Did not receive diet effects' means that the F1 fly was not related to the F0 fly and therefore the F2 fly was unable to pass on the diet effects through them. (*b*) Experimental design. The F0 generation was administered either higher (20% of overall solution) or lower (2.5%) relative sucrose in adulthood, and kept on this diet in sex-specific cohorts for 6 days as virgins before a subsequent 3 day cohabitation (on common garden media—an intermediate sucrose content of 5%) that allowed mating to occur. Male and female F0 flies were combined in all possible diet combinations. The F1 generation was reared, maintained (6 days again), and cohabited (3 days) on common garden media. The F2 generation was reared from egg-to-adulthood on common garden media, and then challenged as virgin adults with either the higher or lower sucrose such that their diet either matched or mismatched one or both of their grandparents (F0). Sample size for F0 was 1280 flies (640 of each sex); for the F2 sample size was 4080 flies (2040 of each sex). Figure made with BioRender.

Here, therefore, we experimentally tested the capacity for dietary sucrose variation among male and female *D. melanogaster* to trigger transgenerational effects on components of life-history (primarily lifespan and reproduction) and physiology in their grandoffspring. Flies were administered one of two diets that varied in the concentration of sucrose (2.5% or 20% sucrose). The diets were administered using a full factorial design: males and females were each assigned to one of the two diets prior to reproduction, and then their grandsons and granddaughters were administered the same dietary treatments. All grandparent × grandoffspring dietary combinations were represented in both males and females in each generation, resulting in diet combinations in which grandoffspring were either matched or mismatched with one or both grandparents (figure 1*a*,*b*).

## 2. Methods

### (a) Study species and generating experimental flies

We sourced flies from Dahomey, a large laboratory population of *D. melanogaster*, originally sourced in 1970 from Benin West Africa [32]. The flies have been maintained in large population cages, with overlapping generations in the Piper laboratory, Monash University, Australia, since 2017, and prior to that in the Partridge laboratory, University College London [33]. Prior to the beginning of the experiment, we collected approximately 3000 eggs from the cages, and distributed them into 250 ml bottles containing 70 ml of food. Food comprised 5% sucrose (50 g sucrose, 100 g yeast, 10 g agar per litre) solution with an estimated protein to carbohydrate (P : C) ratio of 1 : 1.9, and 480.9 kcal l^−1^ (see electronic supplementary material, figure S4 for further diet details). Every generation (for seven generations), adult flies eclosing from multiple bottles were admixed prior to redistributing the flies across new bottles. To control for potential sources of variation in their environment, during these seven generations we strictly controlled both the age of flies at the time of ovipositioning—all flies were within 24 h of eclosion into adulthood when producing the eggs that propagated the subsequent generation, and their population density was 300–320 adult flies within each bottle in each generation.

### (b) Dietary treatments

The diet media we used consists of sucrose, autolysed brewer's yeast powder (sourced from MP Biomedicals SKU 02903312-CF), and agar (grade J3 from Gelita Australia), as well as preservatives—propionic acid, and Nipagin. We prepared two dietary treatments, differing in relative sucrose concentration; 2.5% sucrose (that we refer to as a lower sucrose treatment relative to the 5% concentration usually provided to the population of flies used in this experiment), and 20% sucrose (that we refer to as a higher sucrose treatment) of overall food solution. The 2.5% sucrose diet contained 25 g of sucrose, 100 g of yeast and 10 g of agar per litre of food prepared, with an estimated P : C ratio of 1 : 1.4 and 380.9 kcal per litre of food. The 20% sucrose treatment contained 200 g of sucrose, 100 g of yeast, and 10 g of agar per litre of food prepared, with an estimated P : C ratio of 1 : 5.3 and 1080.9 kcal per litre of food. The diets thus differed not only in sucrose concentration, but overall macronutrient balance and their total caloric content.

We used varying levels of sucrose in our experiments because it is ecologically relevant to do so, as fruit flies in a natural environment can experience both spatial and temporal heterogeneity in their diet, i.e. depending on what food is available to them at that time in that place. Fruit flies usually feed on rotting fruits, which depending upon the type of fruit and varying levels of decomposition, will produce differing levels of sucrose (but very low levels of fats) [34]. Additionally, the higher sucrose concentration was selected based on preliminary experiments that we conducted (see supplementary material in Camilleri *et al.*, [11]), and which caused flies to accumulate more body fat (measured by measuring whole-body triglyceride levels), than diets of regular sugar, consistent with results from previous work in *D. melanogaster* [11,25,35]. A total sugar concentration of over 20% tends to result in flies that are too unhealthy to conduct a transgenerational experiment with, i.e. flies either are low in fertility, die before they have a chance to mate, or their offspring are not viable enough to experiment on cross-generationally. It is also estimated that the percentage of overall daily caloric needs from added sugars in the average American diet could be around 13–20% [36] in children, and as high as 57% in adults [37], and those added sugars are often consumed in the form of ultra-processed foods, which are also usually made up of around 20% added sugar [37], therefore our choice of 20% sucrose for fly food is a fair (and conservative) analogue sugar content in a western diet. All diets contained 3 ml l^−1^ of propionic acid and 30 ml l^−1^ of a Nipagin solution (100 g l^−1^ methyl 4-hydroxybenzoate in 95% ethanol) and were cooked according to the protocol described in Bass *et al.* [38]. Each vial is 40 ml in volume, and contained 7 ml of food.

### (c) Experimental design

Male and female virgin flies were assigned to one of two of the dietary treatments prior to mating (we refer to this generation of flies as F0), and then the grandoffspring produced (F2 generation) were also assigned to one of the two treatments. All possible combinations of grandam × grandsire × grandoffspring diet treatment were represented (= 2 × 2 × 2 = 8 combinations). Specifically, we collected 1280 flies of the F0 generation as virgins and placed them onto either the high sucrose (20%) or the low sucrose (2.5%) diets for the first 6 days of their adult life. They were kept in vials of 10 flies across 64 vial replicates per treatment, and per sex (high sucrose: 32 vials of males and 32 vials of females; low sucrose, 32 vials of males and 32 vials of females, 128 vials in total; 1280 flies, 640 of each sex). They were kept in their respective sexes. We transferred flies to vials containing fresh food of the designated diet every 48 h during this 6 day period. At day 6, we randomly sampled six vials from each treatment, and snap froze (using liquid nitrogen) the flies of these vials, storing them at −80°C for subsequent measures of triglyceride levels. Cohorts of flies in the remaining vials then entered a cohabitation phase to enable female and male F0 flies to mate. Cohorts of males and female flies were combined, in vials of 10 pairs, in each of all four possible diet combinations: lower sucrose females × lower sucrose males; higher sucrose females × higher sucrose males; lower sucrose females × higher sucrose males; higher sucrose females × lower sucrose males. During this phase, flies cohabited for 96 h. They were transferred to a new vial with fresh food of standard 5% sucrose diet every 24 h during this time.

The vials from the 6 day old F0 flies (i.e. the vials from day 1 of the 96 h cohabitation phase) were retained, and the eggs that had been laid by females of the respective vials were trimmed to 80 per vial by removing excess eggs with a spatula. The remaining eggs were left to develop into adult offspring over 10 days at 25°C (on a 12 : 12 light/dark cycle in a temperature-controlled cabinet; Panasonic MLR-352H-PE incubator). These adult flies constituted the F1 offspring in the experiment, and F1 flies developed on standard 5% sucrose media. We collected 2080 virgin F1 flies from each of the four combinations of parental diet treatments, and placed them in sex-specific cohorts of 10 individuals per vial, on standard 5% sucrose media for 6 days. We then allowed these F1 males and F1 females to cohabit and mate with male or female *tester* flies (creating 10 pairs per vial). The tester flies were collected from the same Dahomey stock population (but not subjected to a dietary sucrosetreatment) to create the F2 generation. The diet treatments applied to the F0 flies were thus transferred to the F2 generation via either F1 males or F2 females, but never through both sexes. The F1 flies were 6 days of adult age when laying the eggs that produced the F2 generation. We then collected virgin F2 flies (the grandoffspring of the F0 flies) from each of the four combinations of F0 diet treatments (per sex), and placed them in their respective sexes in vials of 10 flies, across 102 vial replicates per diet treatment per sex (4080 flies, 2040 male, 2040 female). We assigned these F2 flies, produced by each dietary treatment combination of F0 flies, to either the lower sucrose or higher sucrose diet. At day 6 of adulthood, we snap froze F2 flies of six randomly chosen vials per grandam × grandsire × grandoffspring combination. On the same day, 10 virgin focal F2 flies of each grandam × grandsire × grandoffspring combination and each sex were placed together with 10 age-matched tester flies of the opposite sex from the Dahomey population, entering into a cohabitation phase of 96 h (during which time the number of eggs laid by females of each vial was assessed). After 96 h flies were separated again into their respective sexes (in vials of 20 experimental flies of the same sex), and assigned back onto either the lower sucrose or higher sucrose diets that they had been on prior to cohabitation, and a lifespan assay carried out.

### (d) Lifespan

We scored the lifespan of experimental flies of the F2 generation. Each vial in the assay commenced with 20 same-sex flies in each, and we included 10 vial replicates per treatment (grandam × grandsire × grandoffspring) (3400 flies total, the original amount collected, minus the snap frozen samples). The number of dead flies per vial was scored three times per week (Monday, Wednesday, Friday), and surviving flies at each check transferred to vials with fresh food of the assigned diet treatment—until all flies were deceased. During the lifespan assay, vials were stored in boxes (of 85 vials per box) that were moved to randomized locations in a (25°C) control temperature cabinet every Monday, Wednesday and Friday to decrease the potential for confounding effects of extraneous sources of environmental variation within the cabinet from affecting the results.

### (e) Fecundity

We measured the egg output of female flies from generations F0 and F2 at 8 days following eclosion, as a proxy of female fecundity. On day 8, female flies oviposited for a 22 h period, and were then transferred to fresh vials. Day 8 was selected because fecundity over 24 h at this age has been shown to correlate with total lifetime fecundity of females in this Dahomey population and early, short-term measures of reproduction of between 1 and 7 days can be used to accurately predict total lifelong fecundity in *D. melanogaster* [39]*.* Moreover, previous data shows that varying the range of sucrose concentrations did not alter the timing of reproductive peaks between treatments [38]. For the F0 generation, we counted eggs from vials, each containing 10 female flies that had been mated with 10 male flies, across two different sucrose levels (2.5% and 20% sucrose), and four different mate diet combinations (dam diet × sire diet combinations). For the F2 generation, we counted eggs from each grandam × grandsire × grandoffspring dietary treatment combination; each combination was represented by 10 vial replicates, each containing 10 focal females (females from the experiment) combined with 10 tester male flies. Additionally, we counted the number of adult flies that eclosed within 10 days from the eggs laid by F2 females (a composite measure of clutch viability and juvenile developmental speed). F2 females cohabited and mated with age-matched tester males of the Dahomey population, for 24 h at 6 days of life, and the vials containing these eggs were left to develop into adult offspring, for 10 days at 25°C; 12 : 12 light/dark cycle in a temperature-controlled cabinet (Panasonic MLR-352H-PE incubator).

### (f) Lipids and protein

Whole-body triglyceride levels (referred to below as TAG) were measured in adult flies from the F2 generation (6 days of adult age, corresponding with 6 days of exposure to the relevant F2 dietary treatment, prior to mating) and normalized to protein content (full protocols reported in the electronic supplementary material). Three biological replicates per treatment level, with three technical replicates per biological replicate were used. Five female flies and eight male flies, respectively, were used for each biological replicate in the assay, to standardize weight for each sample. We chose to measure whole-body triglycerides in flies as a proxy measure of overall accumulation of body fat under the differing diet treatments, as previous studies have shown increasing sucrose levels in fruit fly diets tend to also increase triglycerides [35].

### (g) Statistical analyses

We used R (Version 3.6.1) and RStudio (Version 1.2.1335) [40] for statistical analyses. To test the effects of F0 female diet, F0 male diet, F2 diet, and sex on F2 lifespan, TAG, and offspring production, we fitted linear mixed effects models (separate models per trait), using the R package lme4 (v. 1.1–27.1) [41]. We use the term lifespan to denote the age of recorded death for each individual fly within a margin of 72 h (for example, a lifespan of 30 days indicates that a fly died between 27–30 days post eclosion). To test the effects of grandmaternal diet, grandpaternal diet, grandoffspring diet, and sex on female fecundity, we fit a general linear model to the egg output data for both generations.

We included F0 male, F0 female, F2 diets, and F2 sex as fixed effects in each model, respectively, exploring all possible interactions between these factors. We included the vial identification number as a random effect in the lifespan models. The fecundity models only included one observation per vial because we counted total eggs per vial, and divided by the number of females in the vial (approx. 10 females) therefore, no random effects were included in this model. The viability of the F2 grandoffspring (how many F3 eclosed) included ‘Counter’ (the person who counted the flies) as a random effect. The model that investigated the F2 whole-body TAG included, plate reading replicate, technical replicate and vial ID as random effects.

We used log-likelihood ratio tests that reduce the full model, via the sequential removal of highest order interactions that did not (significantly) change the deviance of the model, using a *p*-value significance level of less than 0.05. The final reduced models (except fecundity measures) were fit by restricted maximum likelihood, applying type III ANOVA with Kenwood–Roger's *F*-test and approximation of denominator degrees of freedom. We used sum to zero constraints in all models, and we visually inspected diagnostic plots for the linear mixed effect models, to ensure that the assumptions of normality and equal variances were met.

## 3. Results

### (a) Direct and indirect effects of dietary sucrose on grandoffspring lifespan are sex-specific

**Figure 2.**
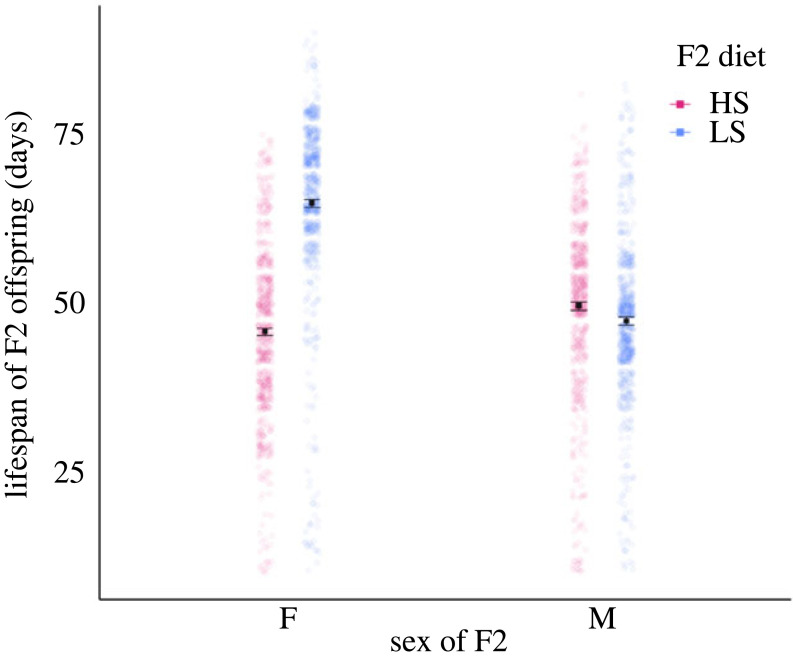
Direct effects of dietary sucrose on the lifespan (plots show estimated marginal means (emmeans) ± standard error, calculated at the vial level, electronic supplementary material, table S1, and data points are raw data, each data point is an individual fly) of F2 granddaughters (F) and grandsons (M). HS indicates a high sucrose diet of 20% (P : C ratio 1 : 5.3), LS indicates a low sucrose diet of 2.5% (P : C ratio 1 : 1.4).

The diets of the grandoffspring (F2) flies conferred direct and sex-specific effects on their lifespan (*F*_1,148_ = 369.80, *p* < 0.001, electronic supplementary material, table S1, figure 2). Female F2 flies assigned to the low sucrose diet lived longer than females or males assigned to any other treatment, and 30% longer than females on the high sucrose diet. Females assigned to a high sucrose diet exhibited the shortest lifespan of any group of flies. In contrast to the large negative effect of high sucrose on female lifespan, high dietary sucrose conferred a moderate increase in male lifespan relative to males assigned to a low sucrose diet (electronic supplementary material, table S1; figure 2).

**Figure 3.**
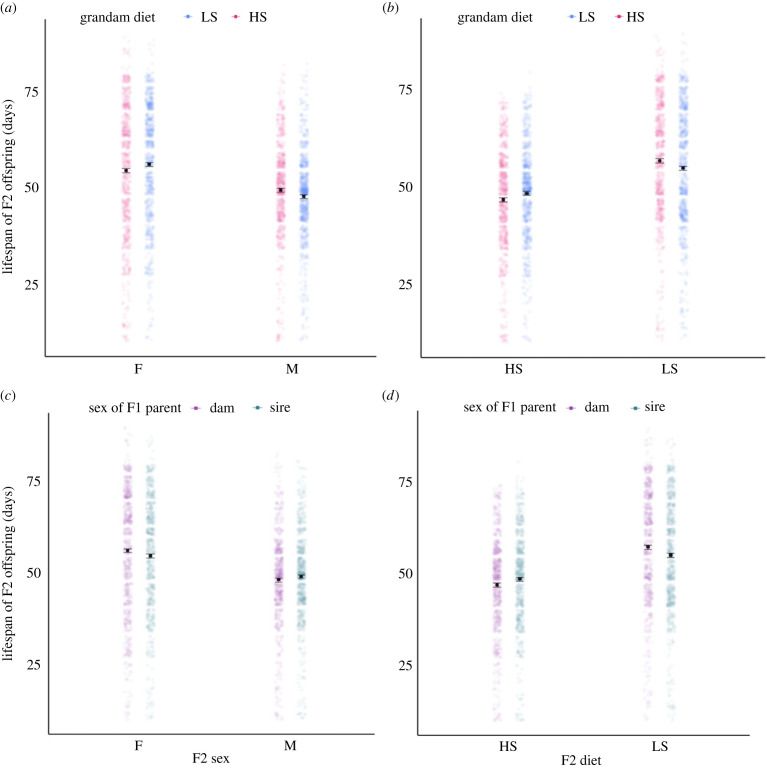
Effects of high sucrose (HS, 20% of overall solution) and low sucrose (LS, 2.5% of overall solution) on F2 lifespan (F = female, M = male). Plots show estimated marginal means, standard error bars, calculated at the vial level (electronic supplementary material Table S1) and data points are raw data, each data point is an individual fly. (*a*) Lifespan of F2 flies (*y*-axis), their F0 grandam's diet (colour), and the F2 sex (*x*-axis), (interaction: grandam diet × F2 sex). (*b*) Lifespan of F2 flies (*y*-axis), their F0 grandam's diet (colour), their diet (*x*-axis), (interaction: grandam diet × F2 diet). (*c*) Lifespan of F2 flies (*y*-axis), the sex of the parental linage that received a diet treatment, (colour), their sex (*x*-axis), (interaction: F1 sex × F2 sex). (*d*) Lifespan of F2 flies (*y*-axis), the sex of the parental linage that received a diet treatment (colour), their diet (*x*-axis), (interaction: F1 sex × F2 diet).

The lifespan of F2 flies was also in part mediated by the diets of their grandmothers, with the pattern of effects differing across F2 males and females (*F*_1,148_ = 9.35, *p* < 0.01, electronic supplementary material, table S2, figure 3*a*). The transgenerational effects of sucrose concentration mimicked the direction of direct effects described above. That is, F2 females descended from grandmaternal lineages assigned to a low sucrose diet lived longer than those descended from high sucrose lineages, while the opposite pattern was observed in F2 males, whereby those descended from high sucrose grandmaternal lineages outlived those from low sucrose lineages (figure 3*a*). Additionally, matching combinations between F0 grandmaternal, and F2 grandoffspring dietary sucrose led to shorter F2 lifespan than mismatched combinations (figure 3*b*). In our experimental design, F0 grandparental flies were manipulated, and F2 phenotypes grandmaternal–grandoffspring dietary sucrose led to shorter F2 lifespan than mismatched combinations (figure 3*b*).

Plots show estimated marginal means (emmeans) ± standard error, calculated at the vial level using R package ‘emmeans’ (v. 1.10.0.).

In our experimental design, grandparental flies were manipulated, and F2 phenotypes measured. This involved transfer of effects across an intermediate generation—the F1 parents. Although the diets of F1 parents were never manipulated, (they received a standard diet of 5% sucrose, an intermediate sucrose content), our experimental design ensured the grandparental (F0) effects were transferred through either male F1 or female F1 flies (but not both simultaneously, figure 1). Thus, we could track whether the sex of the *transferring F1 parents* affected the pattern and direction of the transgenerational effects. Indeed, the interaction between the sex of the F2 flies and the sex of the transferring F1 parents affected F2 lifespan (*F*_1,148_ ***=*** 4.44, *p* < 0.05, electronic supplementary material, table S1); female F2 lived longer if the grandparental dietary treatments were transferred through F1 females, while male F2 lived longer when the effects were transferred through F1 males (figure 3*c*). The sex of the transferring F1 parent flies also moderated the direct effects of the F2 diet on F2 lifespan, electronic supplementary material, table S1, figure 3*d*, *F*_1,148_ = 12.42, *p* < 0.001). F2 flies assigned directly to a high sugar diet lived longer if grandparental dietary treatments (regardless of whether they were low or high in sucrose) were transferred through F1 males rather than through females, while F2 flies assigned to a low sugar diet lived longer if grandparental dietary treatments were transferred through F1 females than males.

### (b) Grandoffspring fecundity, viability and triglycerides are mediated by grandmaternal and grandpaternal diets

#### (i) Fecundity and viability

**Figure 4.**
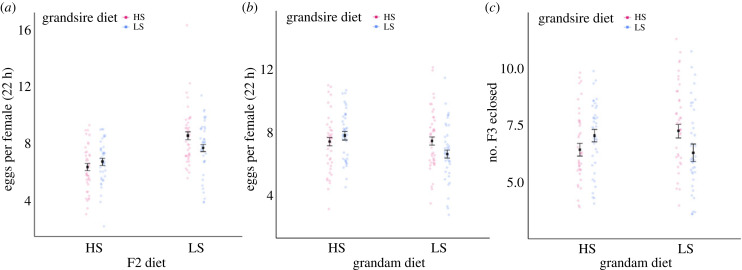
Effects of high sucrose (HS, 20% of overall solution) and low sucrose (LS, 2.5% of overall solution) on female F2 reproductive output. Females oviposited for 22 h to produce eggs. Plots show estimated marginal means, standard error bars (calculated from the models in electronic supplementary material, table S2 and S3) and data points, each data point is the number of eggs or offspring adults produced per female F2. (*a*) Number of eggs laid by F2 flies (*y*-axis), their grandsire's diet (colour), their diet (*x*-axis), (interaction: grandsire diet × F2 diet). (*b*) Number of eggs laid by F2 flies (*y*-axis), their grandsire's diet (colour), their grandam's diet (*x*-axis), (interaction: grandsire diet × grandam diet). (*c*) Number of F3 flies eclosed per vial (*y*-axis), their grandsire's diet (colour), their grandam's diet (*x*-axis), (interaction: grandsire diet × grandam diet).

Direct dietary effects were observed in the F2 generation; F2 females had higher fecundity when ingesting the low sucrose than the high sucrose diet. These direct effects of diet were, however, shaped by the grandpaternal, but not grandmaternal diet (electronic supplementary material, table S2, *F*_1_ = 5.49, *p* < 0.05). Mismatched combinations of grandpaternal–F2 female diet resulted in F2 granddaughters producing more eggs than matched combinations (figure 4*a*). Female F2 fecundity was also shaped by an interaction between the grandmaternal and grandpaternal diets (electronic supplementary material, table S2, figure 4*b*, *F*_1_ = 14.77, *p* < 0.05); F2 females that descended from matched grandmaternal–grandpaternal combinations tended to have lower fecundity than those arising from mismatched combinations, and in particular F2 females descended from grandparents that were each assigned to low sucrose diets exhibited lowest fecundity (figure 4*b*). The reproductive success (as gauged by the number of adult offspring produced) of the F2 females was also shaped by a similar interaction between grandmaternal and grandpaternal diet, whereby the clutch size was lower for F2 females descended from matched, relative to mismatched, combinations of grandmaternal–grandpaternal diet (electronic supplementary material, table S3, figure 4*c*, *F*_1_ = 5.25, *p* < 0.05).

### (c) Triglyceride levels

**Figure 5.**
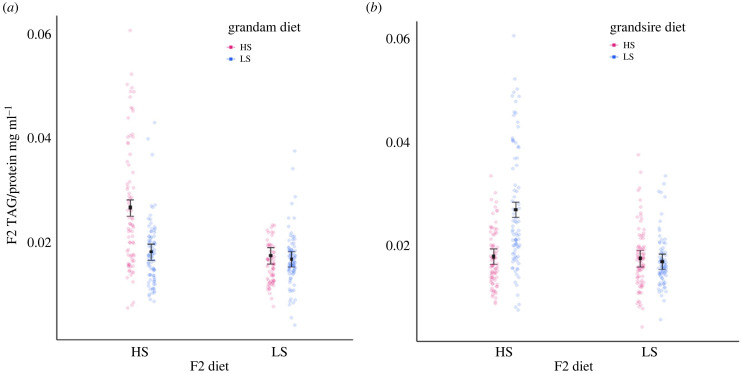
Effects of high sucrose (HS, 20% of overall solution) and low sucrose (LS, 2.5% of overall solution) on F2 whole body triglyceride (TAG) levels divided by their whole body protein levels, per fly. Plots show estimated marginal means, standard error bars, (calculated from the model, electronic supplementary material, table S4) and data points, each data point is the amount of TAG divided by the amount of protein for each group of flies—five females and eight males. (*a*) F2 TAG per fly (*y*-axis), their grandam's diet (colour), their diet (*x*-axis), (interaction: grandam diet × F2 diet). (*b*) F2 TAG per fly (*y*-axis), their grandsire's diet (colour), their diet (*x*-axis), (interaction: grandsire diet × F2 diet).

An interaction between the diet of F2 offspring and the grandmaternal diet affected the triglyceride level of the F2 flies (electronic supplementary material, table S4, figure 5*a*, *F*_1,98_ = 8.56, *p* < 0.01). F2 flies fed high sucrose diets that descended from grandmothers assigned to high sucrose, exhibited much higher triglyceride levels than F2 flies from any other combination of grandmaternal–F2 offspring diet (figure 5*a*). Similarly, the interaction between F2 diet and grandpaternal diet shaped triglyceride level; however in this case, F2 offspring assigned to a high sucrose diet and descended from grandfathers assigned to low sucrose, exhibited much higher triglyceride levels than any other combination of grandpaternal–F2 diet (electronic supplementary material, table S4, figure 5*b*, *F*_1,98_ = 12.75, *p* < 0.001).

### (d) No direct effect of dietary sucrose on female F0 fecundity

Neither the male, nor female diet, affected egg output of the F0 females (electronic supplementary material, table S5).

## 4. Discussion

Here, we aimed to determine whether dietary-mediated parental effects are inherited across multiple generations, and if so, whether they are transferred primarily through maternal or paternal lineages or hinge on interactions between both. We also aimed to investigate whether diet-mediated transgenerational effects act to enhance or depress offspring life history, and whether those effects act in the same way across offspring sexes.

First, we found that diet-mediated parental effects are detectable across multiple generations (to the F2), and moreover that these effects differ depending on whether they were transferred through the maternal or paternal lineage. We found complex interactions and sex-specific transgenerational effects. Grandmaternal diet exerted opposing effects on offspring lifespan depending on offspring sex and diet. Notably, these dietary-mediated effects were observed in both direct and indirect (i.e. directly on the F0 grandparents) and indirectly (i.e. transgenerational effects seen in the F2 grandoffspring) contexts. Moreover, the dietary-mediated transgenerational effects on lifespan mimicked the observed direct effects for each sex: a low sucrose grandmaternal diet conferred elevated F2 female lifespan, but decreased male F2 lifespan, relative to a high sucrose grandmaternal diet. Next, we revealed that diet-induced transgenerational effects act to enhance or decrease offspring life history depending on these specific combinations of grandparental and grandoffspring diets, and between grandmaternal and grandpaternal diets. All of these interactions exhibited a similar pattern—a mismatch in diet enhanced trait expression in the grandoffspring. These results highlight the inherent complexity in the nature of the transgenerational effects. The effects are generally sex-specific, contingent on complex interactions between each grandparent, and between grandparent–grandoffspring, combinations and furthermore, affected by the sex of the transferring F1 parent.

Studies investigating sex-specificity of transgenerational effects across a range of taxa have observed instances in which environmental modification such as dietary challenges, presence of predators, or behaviour-modifying drugs of the grandparental environment triggered sex-specific effects on grandoffspring phenotype. Intriguingly, in these cases, transgenerational effects tend to manifest in the opposite sex to that subjected to the grandparental treatment; that is, modification of the grandmaternal environment may enhance or inhibit trait expression (offspring life-history performance) specifically among grandsons, or conversely, modification to the grandpaternal environment may enhance or inhibit trait expression specifically among granddaughters [10,27,29–31]. Our findings are consistent with previous research, in that we revealed opposing directions of sucrose-mediated grandmaternal effects in each of the sexes.

However, previous studies of dietary-mediated transgenerational effects have tended to focus on changes in metabolite profiles and physiology across generations, rather than on changes to expression of life-history traits [25,26]. Investigations into life-history traits are imperative in assessing the adaptive significance of transgenerational effects on offspring, given the close link between these traits and lifetime fitness [5]. Moreover, the experimental designs in previous studies typically have not had the requisite power to partition relative influences of (grand)maternal and (grand)paternal effects on transgenerational phenotypes, nor the factorial design required to determine whether environmental mismatches across generations tends to enhance or depress performance [9]. Our findings result from use of a fully factorial design that allowed us to uncover unexpected levels of complexity in the nature of the transgenerational effects.

We revealed that sex differences in the magnitude of transgenerational effects (the effect transmitted from dietary-treated F0 flies to F2 flies) are dependent on the sex of the transferring F1 parent. We also observed that the outcomes of transgenerational effects depend on interactions between the diets of the grandparents and those of the grandoffspring; dietary mismatching across generations tends to enhance lifespan (mediated by a grandmaternal-by-grandoffspring interaction) and fecundity (mediated by a grandpaternal-by-granddaughter diet interaction). Whether or not these effects are mediated by underlying triglyceride levels of the experimental flies remains unclear; yet one pattern was notable, suggestive of a possible transgenerational link between physiology and lifespan. F2 offspring assigned to a high sucrose treatment, and descended from high sucrose grandmaternal lineages exhibited the highest triglyceride levels and the shortest lifespans. Higher overall triglyceride levels indicate a higher fat to protein body ratio which previous studies have identified as a significant indicator of metabolic health.

Our finding that dietary mismatching (between both grandparents and between grandparents and grandoffspring) tends to enhance trait expression adds new insight to studies investigating transgenerational effects of diet, and of transgenerational effects of environmental change more generally. The traditional prediction is that a matching of environment between grandparents and grandoffspring may augment offspring fitness-related traits because the matching environments may allow parents to prime offspring to cope with environments that their parents faced (anticipatory effects). The evidence for anticipatory effects across contexts and taxa is, however, mixed and weak [9,28], and many studies that have leveraged experimental designs with the power to test for these effects have primarily focused on intergenerational effects (from F0–F1) [28], with very few studies classified as transgenerational where grandoffspring should have no direct experience of the grandparental environment, which in flies is F2+, but for species with a gestational period may be F3+ [42]. Our study generally revealed patterns that were contrary to the predicted pattern—dietary mismatching, rather than matching, between grandparents and F2 offspring tended to augment offspring performance. This raises the question of whether cross-generational dietary mismatching may be a general phenomenon that may extend beyond the diets used in our study; the focus in our study was on varied sucrose levels, but future studies should test whether a wider range of diet challenges that also vary in their protein concentrations would yield similar results.

Two recent studies shed some light on this question. Deas *et al*. [43] manipulated dietary quality across three generations (F0 to F2) in *D. melanogaster*, providing flies of each generation with a ‘rich’ diet (rich in calories and supplemented with yeast) or a poor diet (calorie diluted, with no yeast supplementation) in all combinations, and then measuring phenotypic expression in the grandoffspring (F2). They reported that a mismatch between the diet quality (poor versus good diet) of grandams and granddaughters led to a faster development time in the pupal stage of the granddaughters, but this effect did not hold for the entire development time [43]. That study also focused on females, and therefore was not able to capture sex-specificity in any generation. On the other hand, Camilleri *et al*. [11] tested effects of dietary mismatching of F0 flies and their F1 offspring, manipulating the diets of parents of each sex and their offspring, and utilizing the same sucrose diets as we use in the current study. They found that dietary mismatching between parents and F1 offspring led to an increase in lifespan, and fecundity of the F1 offspring [11]. Here, we advance these findings by demonstrating that these effects of dietary mismatch are carried over for multiple generations and are also dependent on the sex of F1 lineage. As the effects are unambiguously transgenerational (extending from F0 to F2), we suggest they are less likely to result from differences in condition of the grandparental flies, and more likely to be mediated via epigenetic mechanisms. Yet, a unifying picture of which nutritional circumstances in the parental generation might be adaptive for offspring remains elusive. We certainly see that a parental (or in this case a grandparental) diet confers effects on offspring life-history traits, and therefore affects their adaptive capacity in some way, but we do not yet know if a parental nutritional effects can be consistently adaptive to their offspring. Rather, we may be seeing the interaction between the grandparental and offspring plasticity in response to their individual diets. This is part of a larger question about the adaptive capacity of plasticity which currently does not itself have a unifying framework (that reliable predictions can be made upon) [44] i.e. when does a plastic effect become an adaption? Therefore, making such predictions about the adaptive nature of cross-generational plasticity within a nutritional context is even more difficult.

## 5. Conclusion

In sum, our work uncovers dietary-mediated transgenerational effects that are on one hand remarkably consistent across generations—transgenerational effects of sucrose tended to mimic the direct effects—but on the other hand are complex and mediated by grandsire–grandam, and grandparent–grandoffspring interactions. We have also extended previous work to demonstrate that dietary mismatching across generations tends to augment the transgenerational phenotype in a manner that is unlikely to be directly linked to condition-dependence. We suggest that future work should focus on uncovering the ecological and evolutionary significance of these results, and the underpinning mechanisms that regulate these complex interactions. We suggest that a process in which transgenerational dietary mismatching promotes fitness of future generations could buffer populations from future changes in aspects of the environment and be particularly adaptive for species that live and depend on ephemeral resources for their source of nutrients. If this is the case, then populations evolving in fluctuating environments may be more likely to evolve mechanisms that promote the fitness of offspring encountering novel environments.

## Data Availability

Data and code are published via Dryad: https://doi.org/10.5061/dryad.zkh1893h1 [45]. Supplementary material is available online [46].
